# Combining stress inoculation with virtual reality simulation training of malignant hyperthermia

**DOI:** 10.1186/s41077-024-00308-0

**Published:** 2024-08-16

**Authors:** Erin E Blanchard, Zina Trost, Michelle R Brown, Corey Shum, Merrick Meese

**Affiliations:** 1 Department of Health Services Administration, University of Alabama at Birmingham, Birmingham, AL USA; 2 Department of Psychological and Brain Sciences, Texas A&M University, College Station, TX USA; 3 Immersive Experience Laboratories, Birmingham, AL USA; 4 Department of Anesthesiology, Vanderbilt University Medical Center, Nashville, TN USA

**Keywords:** Virtual reality simulation, Stress, Malignant hyperthermia, Medical students

## Abstract

**Background:**

Limited research has been conducted on how healthcare simulation can mitigate clinician stress. Stress exposure training (SET) has been shown to decrease stress’s impact on performance. Combining SET with virtual reality (VR) simulation training has not yet been explored in the context of stress inoculation. The primary purpose of this pilot study was to determine if a VR module could induce stress. The secondary purpose was to determine if repeated exposure to stressors could decrease stress response in a simulated environment.

**Methods:**

Medical students were recruited to partake in VR simulation modules aimed at treatment of malignant hyperthermia (MH). Those in the SET group were exposed to stressful stimuli during training modules, while those in the Control group were not. Both groups then completed a Test Module with the presence of stressful stimuli. Objective and subjective indicators of stress were measured after each module.

**Results:**

Both groups indicated increases in perceived stress and module stressfulness after Training Module 1 and decreases after Training Module 2. After the Test Module, the Control group experienced significant elevation in perceived stress (*p* = .05), and the SET group had a significant decrease in perceived module stressfulness (*p* < .05). Both groups had a decrease in perceived competence after Training Module 1 (*p* < .001) and an increase after Training Module 2 (*p* < .001), with the SET group having significant elevation after the Test Module (*p* < .01). Both groups found the VR module to be feasible as a teaching tool. Objectively, the SET group showed an upward trend in electrodermal activity (EDA) from the Tutorial to Test Modules (*p* < .05), with the Control group showing a decrease after Training Module 2 (*p* = .05) and an increase after the Test Module (*p* < .01).

**Conclusions:**

A VR module targeting treatment of MH successfully induced stress and was regarded favorably by participants. Those in the SET group perceived less stress and more competence after the Test Module than those in the Control. Findings suggest that repeated exposure to stressors through VR may desensitize participants from future stress in a simulated environment.

**Supplementary Information:**

The online version contains supplementary material available at 10.1186/s41077-024-00308-0.

## Background

Although research consistently demonstrates that acute and high levels of stress negatively impact technical and non-technical skills in the healthcare setting [1–3], existing healthcare simulation rarely addresses the negative impact of provider stress during intense clinical encounters [4–8]. Stress exposure training (SET), a powerful methodology for teaching important skillsets under predicted environmental stress, has been shown to reduce the negative impact of stress on performance, with little degradation of effect overtime [9–11]. Virtual reality (VR) uses computer technology and a head-mounted display (HMD) to create an interactive, immersive, three-dimensional world in which objects have a spatial presence [12]. While multiple effective simulation methods exist, VR simulation and SET techniques have yet to be combined to address best-practice clinical care decision-making training.

Traditional simulation best practices often require multi-hour live simulations with the presence of several trained experienced facilitators [13], whereas an immersive VR simulation platform has the ability to provide automated (necessary and sufficient) learner feedback based upon in-simulation decision-making and specific learner inputs (e.g., learner visual and spatial tracking). Another critical benefit is in the ability to expose learners to stress-graded clinical scenarios, as articulated by the SET model. In line with cognitive load/performance theories [14], as less cognitive capacity is directed toward stress response and regulation, learners can focus on optimal patient care, concept mastery, skill acquisition, and decision-making. Graded exposure to stressful and complex VR simulations is thus a novel and intuitive application of VR simulation that is expected to mitigate the effects of future stressful encounters while improving perceived performance.

This project sought to leverage advances in VR simulation and SET to address limitations of traditional healthcare simulation platforms in order to concurrently improve medical students’ response to stress when caring for a patient with a medical emergency within a simulated clinical environment. The primary purpose of this pilot study was to determine if a VR module could induce stress. The secondary purpose was to determine if repeated exposure to stressors could decrease stress response in a simulated environment. Simulation has been used for stress inoculation and crisis preparation in both the military [15] and aviation [16] fields. Therefore, we wanted to explore the translation of that to clinical encounters in VR. As such, our hypotheses were as follows: (1) the VR module will induce stress in participants, and (2) repeated exposure to stressful stimuli within the VR module will lessen stress response over time.

## Methods

### Participants

Participants included medical students from the University of Alabama at Birmingham (UAB) School of Medicine. Inclusion criteria included age 18 or older and no prior formal training in malignant hyperthermia. Recruitment was done via email to all medical students and a convenience sample was used. After volunteering, potential participants were screened by a researcher (MM) by phone for study inclusion, which consisted of being a medical student at the institution and being available and willing to participate in person for data collection. After inclusion, participants were divided into two groups: Control and SET. After MM obtained consent, all participants completed the entire study protocol. All procedures were approved by the University of Alabama at Birmingham Institutional Review Board.

### Subjective measures

#### State-Trait Anxiety Inventory: State Items (STAI-State) [17]

Participant state anxiety was assessed through administration of STAI-State items. The STAI-State contains 20 items in which respondents rate their agreement with statements conveying situational anxiety (“I feel nervous;” “I am tense”) on a 4-point Likert scale (1 = not at all; 4 = very much so). Total scores are obtained for the subscale by summing the 20 items. Higher scores indicate greater state anxiety. Internal consistency (Cronbach’s α) for the current sample was *α* = 0.70 initially and 0.80 at follow-up.

#### Current stress

Following each module (see below for module progression), participants provided a response to the question “How do you feel right now?” using a paper and pencil 10-cm Visual Analog Scale (VAS) anchored with the terms “not stressed at all” and “extremely stressed.” Participants made a single mark along the line to represent their appraisals.

#### Perceived module stressfulness

Following each module, participants responded to the question, “How stressful was this last module?” using a VAS anchored with the terms “not at all stressful” to “extremely stressful.”

#### Perceived competence

Following each module, participants responded to the question, “How competent do you feel with the module task?” using a VAS anchored with the terms “not at all competent” to “extremely competent.”

#### Treatment Evaluation Inventory (TEI) [18]

The Treatment Evaluation Inventory (TEI) was administered following the study protocol to assess VR training feasibility/acceptability. The TEI is a standard measure of intervention acceptability and consists of 9 items that assess agreement with positive or negative attitudes toward an intervention on a 1 (strongly disagree) to 5 (strongly agree) scale. Representative items include “I would find this educational tool to be an acceptable way of learning new clinical interventions;” “I like the procedures that may be used in this educational tool;” “I believe this educational tool is likely to be effective.” Items are summed, and scores above 27 on the TEI indicate above moderate acceptability [18]. Internal consistency (Cronbach’s α) for the current sample was *α* = 0.80.

#### Motion sickness

Participants were asked after each module to rank their feelings of motion sickness from 0 (no sickness at all) to 20 (frank sickness).

### Objective measure

#### Electrodermal activity

Electrodermal activity (EDA) has been used widely as a noninvasive measure of emotional or cognitive stress and has been promoted, when combined with subjective stress indicators, as providing a more comprehensive picture of stress within simulation [19]. EDA monitors stress through changes in the electrical conductivity of sweat produced either by the hands or feet [20]. Continuous monitoring of EDA (in micromhos) was collected using a BIOPAC® MP150 data acquisition system connected to an EDA100C amplifier with two LEAD100 electrodes taped to the middle phalanges of the participants’ middle and index fingers. AcqKnowledge®, Version 4 software was used to process the EDA signal and calculate mean levels (in micromhos/minute).

### Malignant hyperthermia crisis virtual reality simulation content and interface

The virtual reality simulation was delivered using a commercial wireless Oculus Quest (Oculus VR, CA, USA) HMD and two controllers, which allowed participants to interact with objects in the virtual world. The simulation facilitated a first-person “embodied” perspective in a virtual reality space, which was a simulated post anesthesia care unit containing one patient. The simulation included patient development of delayed onset malignant hyperthermia (MH) requiring diagnosis and treatment by participants (Image 1). The MH clinical scenario was selected because it is a low-frequency, high-risk clinical diagnosis requiring rapid diagnosis and treatment to promote positive outcomes. Content for the module was heavily influenced by an MH immersive simulation that EB used with anesthesiology residents over multiple years and was modified for VR with input from MM and EB to reflect best practices in simulation design [21]. After each iteration of the module was developed, EB would review the module for usability and alignment with learning objectives, which were to effectively recognize and treat MH in a postoperative patient.

Ecologically salient auditory stress stimuli included regular and random noises, unrelated conversation and instructions communicated from multiple points nearby, expected and unexpected machine communication signals, and the appearance of others engaged in related tasks actively aimed to draw the attention of the learner away from their task sequence. Feedback from trainee and clinician stakeholders was used to grade the stress stimuli into medium and high valence categories. For the current study, auditory stress stimuli were selected to progress from medium to high categories as applicable participants progressed through the training.

**Fig. 1:**
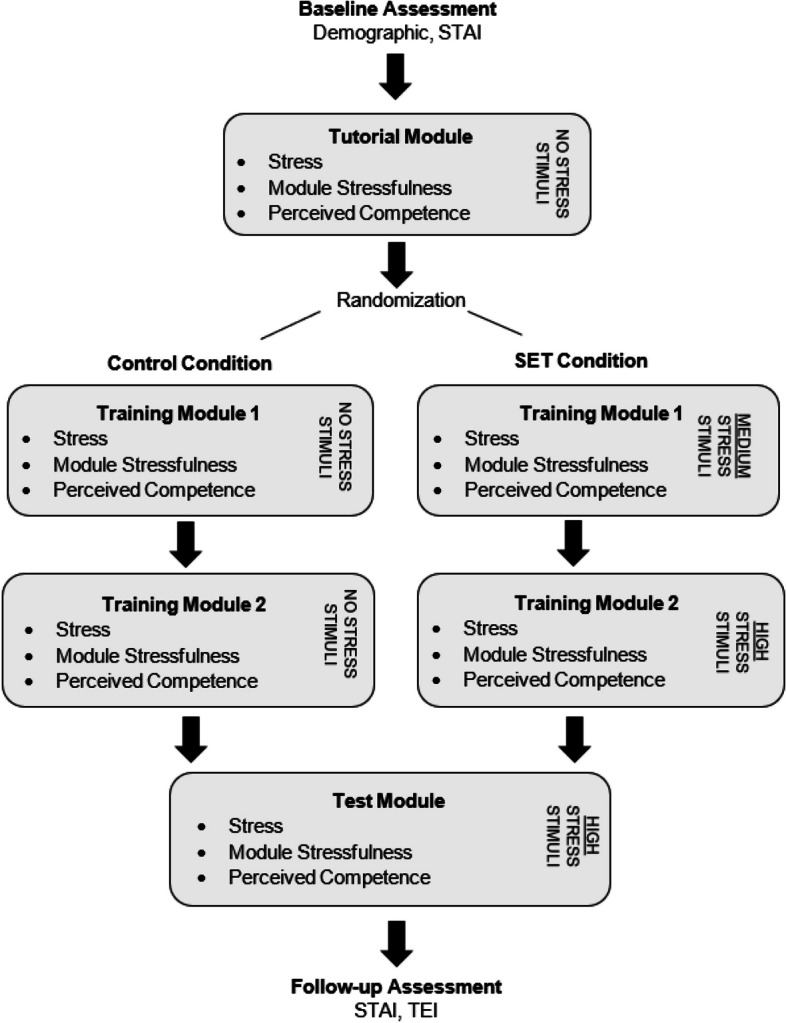
Protocol phases

The full virtual protocol consisted of three sequential phases: Tutorial Phase, Training Phase, and Testing Phase, which each took place in the same virtual scene (Fig. 1). The Tutorial Phase consisted of a single virtual module, during which participants were oriented with the interface, interaction with the virtual environment, mechanics of simulation, and the sequential management of an MH crisis. Each action in the required sequence was presented as a text prompt; the module would continue only upon successful correct action or response. The Tutorial module was identical for participants in the Control and SET condition and did not include stress stimuli. The Training Phase consisted of two successive virtual modules. During the training modules, participants practiced the correct sequence and care choices to manage the MH crisis without prompting; participants were instead provided with real-time feedback regarding incorrect performance (i.e., sequence and selection; see Image 2). Participants in the Control condition completed the sequential training modules without the presence of stress stimuli. For participants in the SET condition, medium- and then high-level stress stimuli were introduced in training modules 1 and 2, respectively. Thus, SET participants received training in a progressively more stressful virtual context. Other than the presence of stress stimuli, the training modules did not differ for the two participant groups.

As part of the single Test Phase module, participants in both the Control and SET conditions were asked to respond to the MH scenario in the presence of a high level of stress stimulation. Participants in the Test module were not provided with prompts or real-time feedback regarding their performance as they completed the action and response sequence from beginning to end.

### Procedure

Upon arrival at the laboratory, participants provided informed consent and completed surveys of demographic/educational information, as well as the STAI-State. Participants were then fitted with physiological equipment and asked to stand quietly to obtain a physiological baseline. Participants were then instructed to don the HMD and complete the Tutorial module and subsequently provide VAS ratings of current stress, perceived stressfulness of the module, and perceived competency. After the Tutorial module and feedback, participants were randomized to the Control or SET conditions and asked to complete the remaining three modules (two Training modules, one Test module). Following each module, participants in both conditions were asked to remove their HDM and provide post-module VAS ratings; participants were likewise asked to stand quietly following each module to return to the physiological baseline. After completion of the Test module, participants provided their post-module ratings and were again asked to complete STAI-State, as well as the TEI as a means of follow-up assessment. Participants were then thanked for their time.

#### Phases of the protocol corresponding to EDA

Key phases of the protocol were defined as (a) end of tutorial module, (b) end of training module 1, (c) end of training module 2, and (d) end of test module. EDA was averaged across phases of the protocol.

### Statistical approach

All analyses were conducted using SPSS Version 22.0. Means, standard deviations, and counts were calculated for relevant study variables. Bivariate correlations were conducted on self-report measures. Two Time × 2 Condition (Control Condition, SET Condition) repeated measures of analyses of variance (RM-ANOVAs) were performed to examine potential changes in anxiety (STAI-State) from pre to post experimental protocol. A series of RM-ANOVAs examined potential change across time and differences between groups in self-reported and physiological indices collected across the four virtual modules. The current sample size reflects the preliminary/pilot nature of the current investigation and is in line with recommendations to approximate a sample size of 12 per group for pilot studies, as the gain in precision of the estimate of variance diminishes once a sample size of 12 is reached [22]. These estimates can then be used to plan a larger confirmatory trial.

## Results

### Sample characteristics

Twenty-eight participants were randomized evenly to either the SET or Control group. One participant from the Control group was subsequently excluded for incomplete data. The remaining participants included 15 men and 12 women, ranging in age from 23 to 35 (*M* = 25.4, SD = 2.95). Of these participants, 17 identified as White, 4 identified as Black or African American, 4 identified as Asian, and 2 identified as Hispanic/Latinx. Eight participants were in their 1st year of medical school, 13 were in their 2nd year, 5 were in their 3rd, and 1 was in their 4th year of medical school. None of the students had received prior training in MH treatment or VR-based clinical simulation training. However, 21 participants indicated that they have previously had “some experience” with non-VR simulation training involving manikins or standardized patients (2 participants indicated no prior experience and 4 participants indicated “a great deal of experience”). Finally, the majority of participants endorsed never having used VR technology (*n* = 10) or using it once (*n* = 11); the remainder used VR technology “a few times” (*n* = 5) or “sometimes” (*n* = 2).

Students spent about 15 to 20 min within the virtual environment. Motion sickness was reported by a small subset of participants, with those in the Control group reporting a marginally higher degree than those in the SET group, *F*(1, 26) = 3.96, *p* = 0.06. However, motion sickness did not necessitate premature cessation of module participation by any participant.

### Subjective measures

#### STAI-State anxiety

Participants in both groups reported a significant decline in self-reported STAI-State anxiety from pre to post experimental protocol, *F*(1, 26) = 10.24, *p* < 0.01. No significant differences were observed between participants in the Control and SET conditions: participant stress, perceived module stressfulness, and perceived competence.

#### Current stress

**Fig. 2:**
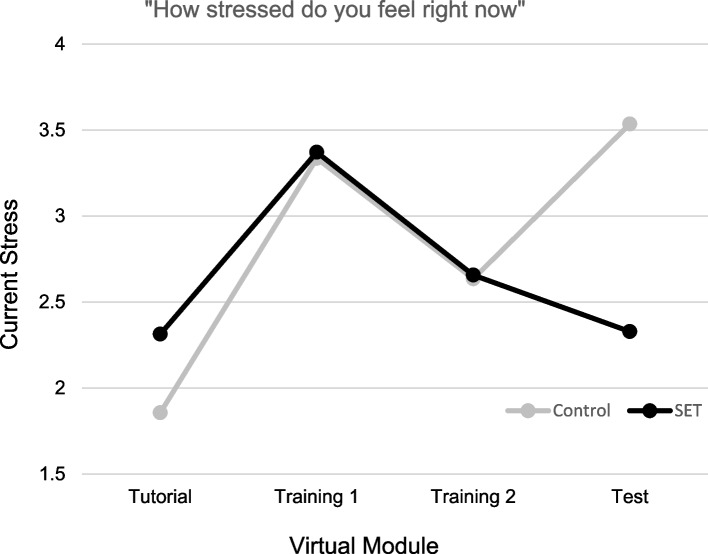
Ratings of current stress. Ratings were using a 10 cm VAS anchored from “not stressed at all” to “extremely stressed”

Figure 2 depicts ratings of current stress for participants in the Control and SET conditions across the four virtual modules. Participants in both conditions endorsed a significant increase in stress from Tutorial module to the first Training module, *F*(1, 26) = 21.85, *p* < 0.001, as well as a significant decrease in stress from the first to the second Training module, *F*(1, 26) = 4.64, *p* < 0.05. A significant Time × Condition interaction was observed for participant stress ratings collected following the second Training module to the final Test module,* F*(1, 26) = 6.38, *p* < 0.05. Specifically, participants in the Control condition reported a significant elevation in stress following the Test module, *F*(1, 26) = 4.64, *p* = 0.05, whereas participants in the SET condition reported a moderate but nonsignificant decline in stress following Test module completion, *F*(1, 26) = 2.16, *p* = 0.10.

#### Module stressfulness

**Fig. 3:**
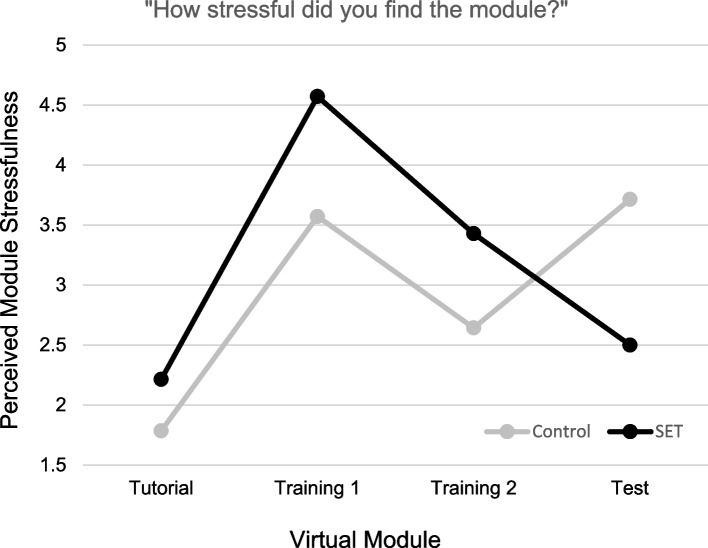
Perceived module stressfulness. Rantings were done on a 10 cm VAS anchored with terms “not at all stressful” to “extremely stressful”

Figure 3 depicts ratings of perceived module stressfulness for participants in the Control and SET conditions across the four virtual modules. As above, participants in both conditions endorsed a significant increase in stress from Tutorial module to the first Training module, *F*(1, 26) = 50.73, *p* < 0.001, as well as a significant decrease in stress from the first to the second Training module, *F*(1, 26) = 8.62, *p* < 0.05. A significant Time × Condition interaction was observed for participant module stressfulness ratings collected following the second Training module to the final Test module,* F*(1, 26) = 8.48, *p* < 0.05. Specifically, participants in the Control condition reported a marginal elevation in perceived module stressfulness following the Test module, *F*(1, 26) = 3.67, *p* < 0.10, whereas participants in the SET condition reported a significant decline in perceived stressfulness following Test module completion, *F*(1, 26) = 5.42, *p* < 0.05.

#### Perceived competence

**Fig. 4:**
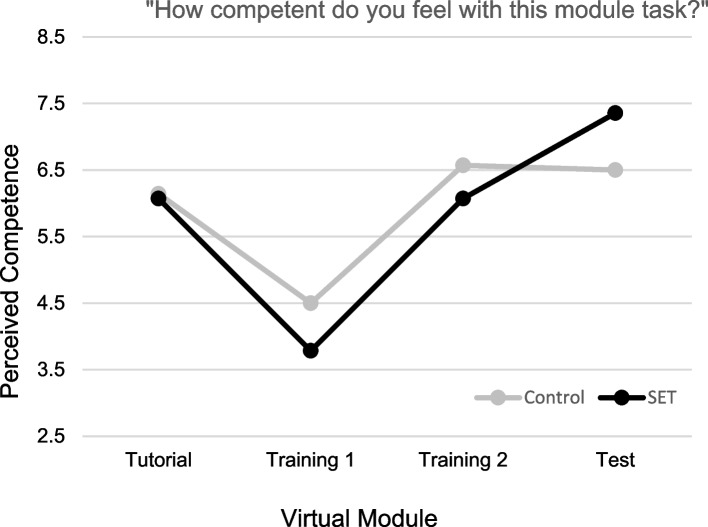
Perceived competence. Ratings were done on a 10 cm VAS anchored with terms “not at all competent” to “extremely competent”

Figure 4 shows ratings of perceived competency with the module material for participants in the Control and SET conditions across the four virtual modules. Participants in both conditions endorsed a significant decrease in perceived competence from Tutorial module to the first Training module, *F*(1, 26) = 54.02, *p* < 0.001, as well as a significant increase in perceived competence from the first to the second Training module, *F*(1, 26) = 21.27 *p* < 0.001. A significant Time × Condition interaction was observed for ratings collected following the second Training module to the final Test module,* F*(1, 26) = 7.56, *p* = 0.01. Specifically, participants in the SET condition reported a significant elevation in perceived competence following the Test module, *F*(1, 26) = 13.16, *p* < 0.01; no significant change in competence ratings was observed for participants in the Control condition.

#### Treatment evaluation inventory

Control and SET conditions did not differ with respect to treatment evaluation, both providing relatively high ratings above the acceptable threshold, *F*(1,25) = 0.39, *p* > 0.05.

### Bivariate correlations of subjective measures

Bivariate correlations between self-reported variables are presented in Table 1. Following the Tutorial module, participant ratings of current stress were significantly positively correlated with perceived module stressfulness (*r* = 0.87) and significantly negatively correlated with perceived competency (*r* =  − 0.59). Likewise, participant ratings of tutorial module stressfulness were significantly negatively correlated with perceived competence (*r* =  − 0.69). Following the first and second training modules, participant ratings of current stress were significantly positively correlated with the perceived stressfulness of that module (*r* = 0.74 and *r* = 0.54, respectively). Following the test module, participants’ current stress was significantly positively associated with test module stressfulness (*r* = 0.62). Module stressfulness was significantly inversely associated with perceived competence (*r* =  − 0.52).

**Table 1:** Associations between study variables

Variable	**1**	**2**	**3**	**4**	**5**	**6**	**7**	**8**	**9**	**10**	**11**	**12**	**13**	**14**	**15**
1. Baseline STAI		.33	.09	.16	.20	− .40^*^	.01	.17	.06	− .06	− .09	.15	− .01	.12	.02
2. Follow-up STAI			− .45^*^	.26	.37	− .15	.18	.34	− .37	.12	.34	− .51^**^	.14	.42^*^	− .50^**^
3. TEI				− .31	− .48^**^	.46^*^	− .01	− .27	.42^*^	− .40^*^	− .27	.47^*^	− .29	− .23	.59^**^
4. Tutorial-stress					.87^**^	− .59^**^	.67^**^	.74^**^	− .26	.44^*^	.33	− .04	.29	.28	− .08
5. Tutorial-module stressfulness						− .69^**^	.60^**^	.70^**^	− .34	.45^*^	.47^*^	− .17	.35	.41^*^	− .26
6. Tutorial-competence							− .13	− .43^*^	.28	− .09	− .20	.13	− .05	− .26	.29
7. Training 1-stress								.74^**^	− .24	.56^**^	.44^*^	.05	.47^*^	.29	.13
8. Training 1-module stressfulness									− .29	.53^**^	.57^**^	− .15	.41^*^	.46^*^	− .07
9. Training 1-competence										− .21	− .18	.63^**^	− .31	− .30	.51^**^
10. Training 2-stress											.54^**^	− .17	.73^**^	.23	− .06
11. Training 2-module stressfulness												− .28	.33	.38^*^	− .02
12. Training 2-competence													− .14	− .37	.77^**^
13. Test-stress														.62^**^	− .21
14. Test-module stressfulness															− .52^**^
15. Test-competence															

Tutorial, ratings collected following the Tutorial 1; Training 1, ratings collected following the first training module; Training 2, ratings collected following the second training module; Test, ratings collected following the test module

*STAI* State-Trait Anxiety Inventory, *TEI* Treatment Evaluation Inventory

*p < .05, **p < .01

Baseline STAI-State was inversely associated with perceived competence following the tutorial module (*r* = *0.4*0). Perceived competence following the first and second training modules and the test module were associated with higher state anxiety at follow up post-test module (*r* =  *− 0.3*7 to − 0.50). Test module stressfulness was likewise associated with greater state anxiety at follow-up (*r* = 0.42).

Higher anxiety scores following the protocol were negatively associated with TEI scores. Higher competency scores following each of the four modules were positively associated with TEI ratings (*r* = 0.40–0.53), whereas perceived stressfulness of the tutorial and second training modules were negatively associated with TEI scores (*r* =  − 0.40 to − 0.47). Finally higher state anxiety at follow up post- test module was also negatively associated with TEI ratings (*r* =  − 0.45).

### Objective measure

#### EDA

**Fig. 5:**
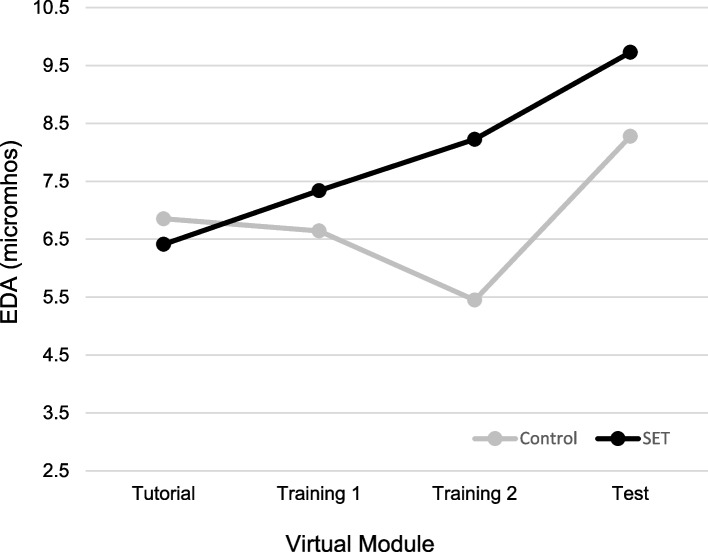
Electrodermal activity

Figure 5 shows changes in EDA across key phases of the study protocol. The current study was only able to collect EDA from a subset of participants. Given the pilot nature of this investigation, findings are presented. However, in light of the small sample sizes, the finding must be interpreted with caution.

Participants in the SET condition showed an overall significant trend in elevation in EDA ratings from the Tutorial to the Test module, *F*(1, 7) = 1.26, *p* < 0.05; an overall significant elevation was observed for participants in the Control condition, *F*(1, 7) = 6.60, *p* = 0.05. A significant interaction, *F*(1, 12) = 6.97, *p* < 0.05 indicated that participants in the Control but not the SET condition showed a significant decline in EDA response from the first to the second Training module, *F*(1, 7) = 6.02, *p* = 0.05. In contrast, Control but not SET participants showed a significant elevation in EDA responses from the second Training module to the Test condition, *F*(1, 7) = 15.71, *p* < 0.01. Elevations in EDA for participants in the SET condition did not reach statistical significance.

## Discussion

The VR MH module was found to cause subjective and objective measures of stress in all learners, validating the stress-inducing intent of the simulation and confirming our hypothesis that the module would cause stress. While participants’ situational anxiety significantly decreased for both groups after the simulations were complete, participants’ self-reported stress differed between modules. Both current stress and perceived module stressfulness increased for Control and SET groups between the Tutorial and Training 1 modules and decreased between Training 1 and Training 2 modules. The Control group had significant current stress and nonsignificant module stressfulness increases after completing the Test module, while the SET group saw a nonsignificant decline in current stress and a significant decrease in module stressfulness once the Test module was finished. This aligns with previous findings of decreasing stress with repeated exposure to similar simulations [23] and increasing stress when encountering a simulated event that may be perceived as more stressful [24].

Findings were mixed with respect to our second hypothesis that exposure to stressful stimuli within the VR module will lessen stress response over time. The SET group showed a trend of significant elevation in EDA from the Tutorial to Test module, indicating that their stress levels were increasing at a steady rate throughout the intervention. Participants in the Control group had a drop in EDA between the first and second Training modules, indicating they were becoming less stressed with repeated exposure to stress-free modules, aligning with previous research on physiologic stress markers during consecutive, similar-stress, immersive simulations [25]. However, from the second Training module to the high-stress Test module, there was a significant increase in EDA in Control group members, suggesting that exposure to stress-free training did not prepare them to deal with the stressors present in the Test module. It is important for facilitators to be cognizant that VR experiences may be overall more stress inducing than traditional didactic or simulation experiences for learners, and appropriate care and considerations should be taken to mitigate this baseline stress. Regarding perceived competence, both groups’ competence levels decreased between the Tutorial and Training 1 modules. This was anticipated as learners transitioned from learning how to navigate the simulation to caring for a patient. The Control group’s competence remained steady throughout the remainder of the modules. The SET group reported increased competence between the Training 2 and Test module. This suggests that, while learners in both groups were initially stressed after the Training module, repeated exposure to stressful stimuli resulted in the SET group becoming less stressed and more confident after the Test module. Additionally, TEI responses demonstrated that, regardless of study group and stress level, participants highly rated the acceptance and feasibility of the VR modules as an educational modality.

Overall anxiety, as measured by STAI-State, decreased after completion of all modules, with no significant difference between groups. When correlated with self-reported stress, the more stressful participants found the modules, the more anxiety they reported. Further, as anxiety increased, perceived competence decreased.

In general, it is important to note that this was a preliminary investigation; given its relatively small sample and the number of comparisons, the current results should be interpreted with caution given the possibility of Type-I error. These preliminary findings should be replicated in larger studies which would allow more rigorous adjustment for multiple comparisons. Our research has several additional limitations. First, although the sample is reflective of the needs of a pilot study, a larger and more diverse sample is needed to draw conclusions. Because all participants were medical students from a single institution, generalizability is also limited. Future research should include clinicians and trainees from multiple disciplines and regions and include longitudinal data collection. Second, although perceived competence was measured, skill level was not assessed. As such, connections between stress exposure and competence can only be hypothesized. An expansion of this work could include tracking the number of correct actions during each module using the VR platform. Third, many measures of stress were obtained by self-report, which can be impacted by many confounding variables. Future studies could include additional objective measures of stress such as heart rate, respiratory rate, and salivary cortisol levels. Finally, only one type of clinical scenario was utilized. It is possible that participants’ responses and reactions were impacted by unfamiliarity with MH treatment guidelines. Future work should include clinical scenarios with which participants are familiar to determine if the embedded stressors were the primary inducers of stress.

## Conclusions

We developed an MH VR simulation that successfully induced stress and was viewed favorably by participants. Those exposed to stressors during training modules perceived themselves to be less stressed during a test module. Those not exposed to stressors during training perceived themselves to be more stressed during the test module. These findings suggest that our MH VR module may inoculate medical students against perceived stress in a simulated environment with repeated exposure. However, caution should be taken in generalizing these findings to other learner groups or simulation scenarios without further testing.

### Supplementary Information

Additional file 1: Image 1. Learner view within the simulation. Image 2. Learner feedback

## Data Availability

The datasets used and/or analyzed during this study are available from the corresponding author on reasonable request.

## Abbreviations

EDA: Electrodermal activity
HMD: Head-mounted display
MH: Malignant hyperthermia
RM-ANOVAs: Repeated measures of analyses of variance
SET: Stress exposure training
STAI-State: State-Trait Anxiety Inventory: State Items
TEI: Treatment Evaluation Inventory
UAB: University of Alabama at Birmingham
VR: Virtual reality
