# Supplementary figures and images for: Combining stress inoculation with virtual reality simulation training of malignant hyperthermia
Source: Adv Simul (Lond). 2024 Aug 16;9:35. doi: 10.1186/s41077-024-00308-0 (PMC11330138; doi:10.1186/s41077-024-00308-0)

Image 1. Learner view within the simulation

**
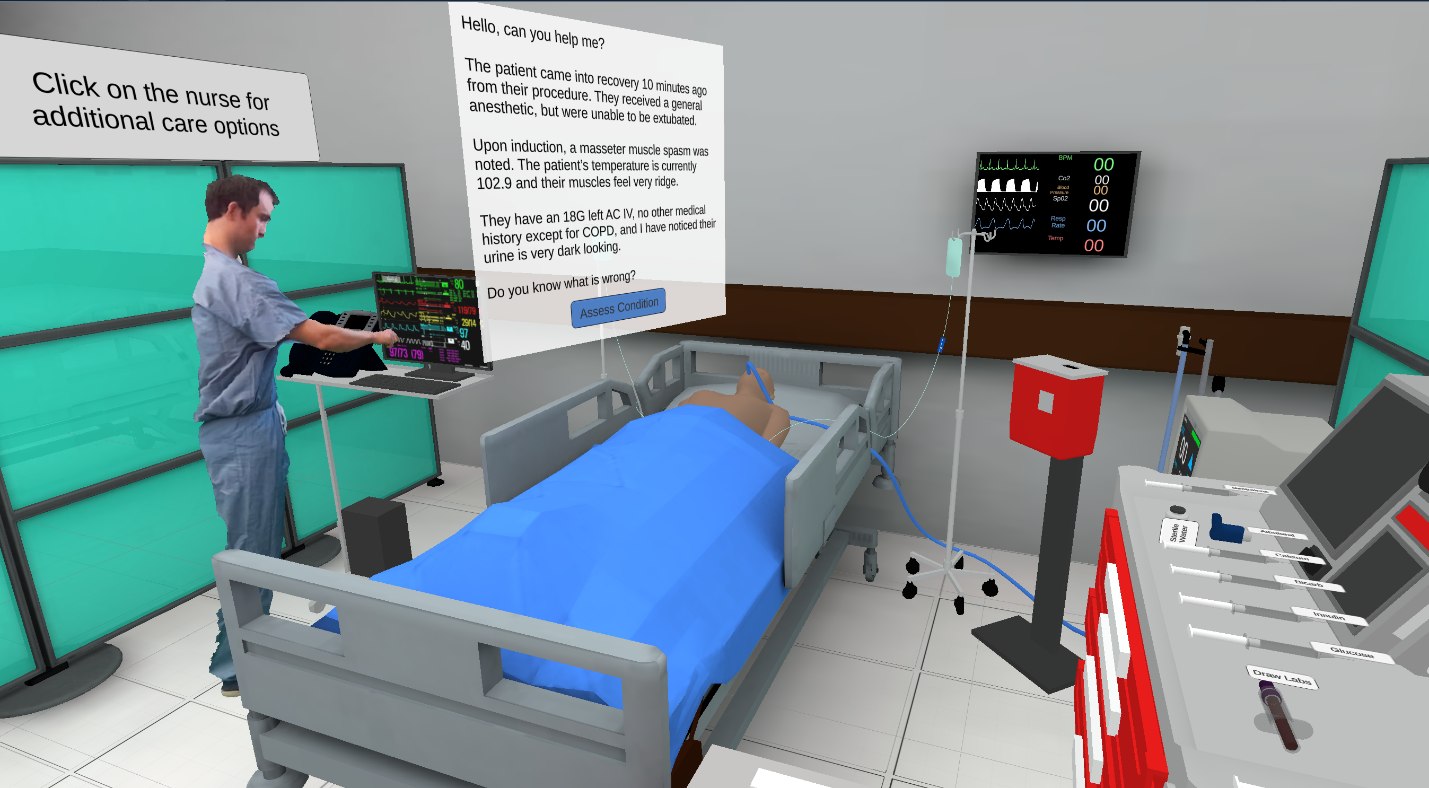
**

Image 2. Learner feedback.


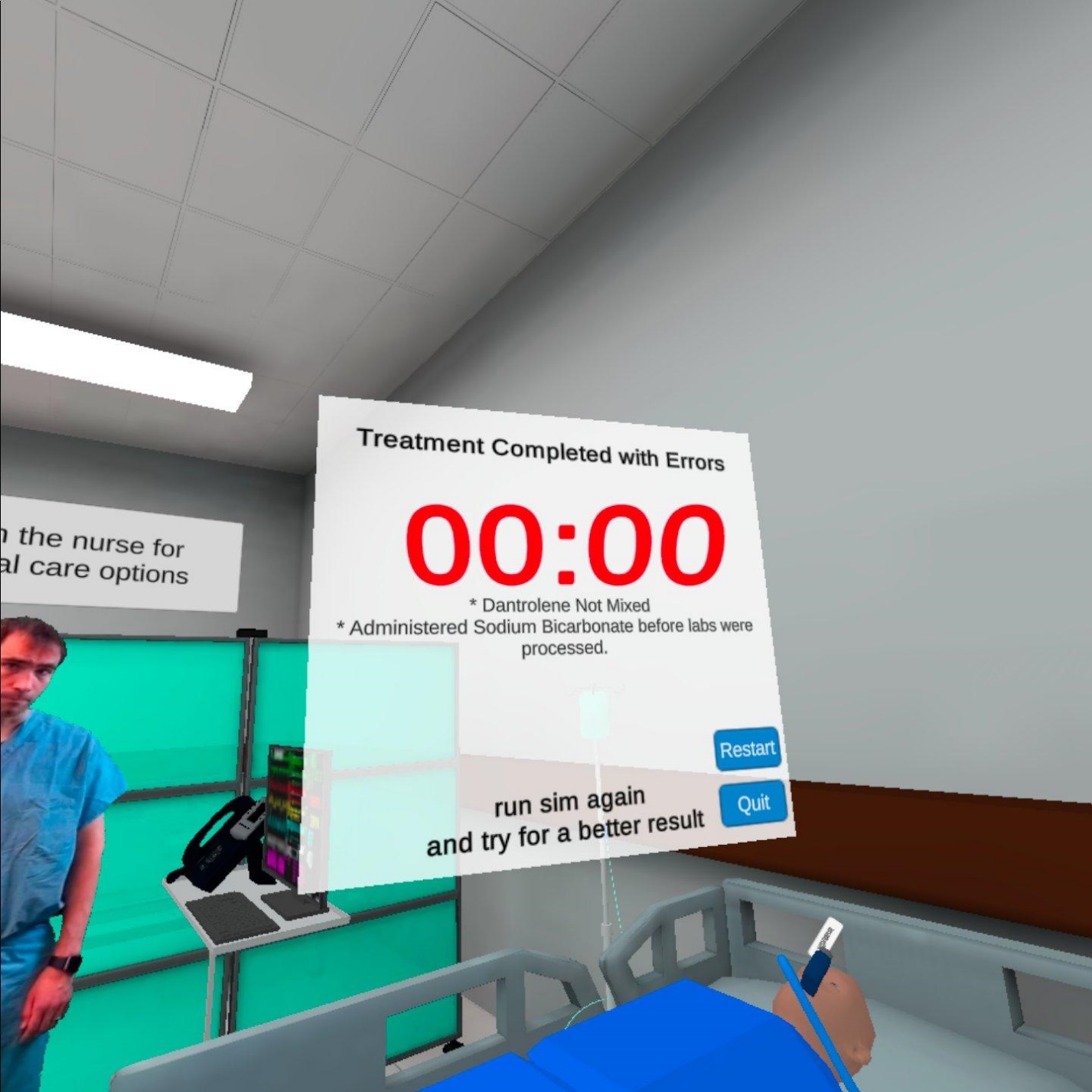

Supplement: Supplementary file 1 — Additional file 1: Image 1. Learner view within the simulation. Image 2. Learner feedback [file 41077_2024_308_MOESM1_ESM.docx]
